# Burden of Elevated Body Mass Index and Its Association With Non-Communicable Diseases in Patients Presenting to an Endocrinology Clinic

**DOI:** 10.7759/cureus.13471

**Published:** 2021-02-21

**Authors:** Saima Ghaus, Tasnim Ahsan, Erum Sohail, Uzma Erum, Wasfa Aijaz

**Affiliations:** 1 Endocrinology, Medicell Institute of Diabetes, Endocrinology & Metabolism, Karachi, PAK; 2 Diabetes and Endocrinology, Jinnah Postgraduate Medical Centre, Karachi, PAK; 3 Endocrinology, Jinnah Postgraduate Medical Centre, Karachi, PAK; 4 Internal Medicine, Liaquat National Hospital, Karachi, PAK; 5 Diabetes and Endocrinology, Medicell Institute of Diabetes, Endocrinology & Metabolism, Karachi, PAK

**Keywords:** obesity, endocrinology, non-communicable diseases, diabetes, hypertension, dyslipidemia, thyroid disorders, autoimmune diseases, fertility, impaired glucose tolerance

## Abstract

Introduction

In the last 45 years, the worldwide rate of obesity has risen by nearly three-folds. Globally, 650 million adults are obese and more than 1.9 billion are overweight. The estimated prevalence of overweight and obesity in Pakistan was found to be 25% and obesity prevalence alone was 10.3% using the Asian-specific body mass index (BMI) criteria. According to the World Health Organization, overweight and obesity increase the risk of non-communicable diseases (NCDs).

Objectives

The aim of this retrospective observational study was to determine the burden of elevated BMI and its association with NCDs among patients presenting to a private endocrinology clinic.

Study design

This was a retrospective observational study conducted at Medicell Institute of Diabetes, Endocrinology & Metabolism (MIDEM), and the study duration was two years.

Methodology

Medical records of the patients who visited MIDEM from January 2017 to December 2018 were reviewed. Patients’ data such as age (in years), gender, height (in cm), and weight (in kg), along with primary complaints and comorbidities were retrieved. BMI was calculated by dividing weight (in kg) by squared height (in m^2^).

Results

A total of 613 records were reviewed. The median age and BMI were 38 years (IQR=18 - 80 years) and 28.8 kg/m^2^ (IQR=24.6-33.05 kg/m^2^),respectively. Out of 613 patients, 10.6% were overweight and 72.6% were obese. Among 510 (83.2%) patients with elevated BMI (≥23 kg/m^2^), the most frequent associated NCDs were dyslipidemia (39.2%), diabetes (32.5%), hypertension (31.4%), thyroid disorders (28.6%), metabolic syndrome (25%), subfertility (14.9%), impaired glucose tolerance (12.7%) and autoimmune diseases (6.9%). On age- and gender-adjusted logistic regression model, the risk of dyslipidemia, hypertension, and diabetes was significantly higher in overweight and obese patients.

Conclusion

This study demonstrated a high prevalence of obesity in patients visiting the endocrinology clinic. Obesity was identified as an independent risk factor for dyslipidemia, hypertension, and diabetes. Future studies are suggested to determine the burden of obesity and establish its association with NCDs in the general population.

## Introduction

Obesity is a complex multifactorial disease, which is a substantial public health problem and has gradually worsened during the past 50 years to epidemic proportions [1]. According to the World Health Organization (WHO), overweight and obesity increase the risk of non-communicable diseases (NCDs). In the last 45 years, the worldwide rate of obesity has risen by nearly three-folds. Globally, 1.9 billion adults, 18 years older or above, were found as overweight and 650 million of these were obese [2]. Elevated body mass index (BMI) is a major public health issue in the first world as well as third-world countries. Around 2.8 million deaths have occurred in the world as a consequence of overweight and obesity [3]. In a study conducted in Pakistan in 2006, the estimated prevalence of overweight and obesity was found to be 25% and obesity prevalence alone was 10.3% using the Asian-specific BMI criteria [4].

Globally, obesity is ranked as the fifth top risk factor for mortality, and it is notable that most of the world’s population lives in countries where being overweight and obesity causes more mortalities than being underweight [2,5]. The NCDs that ensue as a consequence are associated with lesser life expectancy of nearly 5-20 years, depending on severity and other comorbid disorders [6]. Wide range of NCDs that are associated with obesity include cardiovascular diseases (CVDs), type 2 diabetes mellitus (T2DM), hypertension (HTN), dyslipidemia, respiratory problems, subfertility, erectile dysfunction, osteoarthritis, metabolic syndrome, obstructive sleep apnea (OSA), cancer, non-alcoholic fatty liver disease, and psychological issues [7].

Obesity has the potential to cause premature disability and death due as it increases the risk of NCDs. The four leading NCDs, including CVDs, T2DM, chronic respiratory diseases, and cancer, jointly contributed to 78.8% of all NCD deaths worldwide during 2016 [6,8]. Previously NCDs accounted for 52% of total mortalities in South Asia and are predicted to contribute to 72% of total deaths in this region by the end of 2030 [9]. The burden of communicable diseases and NCDs has also doubled in Pakistan as a result of epidemiological transitions, which is a great threat for healthcare systems that are not well prepared to manage the burden of NCDs [10].

In recent times, researchers have emphasized the importance of lifestyle as an essential factor for good health, as 60% of factors for quality of life and health are interrelated to lifestyle, according to the WHO. Millions of people have unhealthy lifestyles and consequently suffer from diseases, disability, and even mortality [11]. Increasing urbanization and emerging technology have led to sedentary behavior and ultimately decreased consumption of healthy food. These facts pushed us to shed the light on the current pattern of BMI in our population [12-14]. To the best of our knowledge, this is the first study from Pakistan that concurrently determined the prevalence of obesity and NCDs among adults visiting an endocrinology clinic. Even though locally generated data have documented the prevalence of obesity and overweight in Pakistan, no substantial studies are available that have investigated the association of multiple NCDs with obesity and overweight. This study was planned to investigate the burden of raised BMI and to establish its association with NCDs among patients presenting to our endocrinology clinic.

## Materials and methods

This retrospective observational study was conducted at Medicell Institute of Diabetes, Endocrinology & Metabolism (MIDEM), which is a specialist endocrinology clinic in Karachi, Pakistan. The study was commenced after approval from the Institutional Review Board. Records of all the patients who visited the clinic for primary or follow-up consultations were reviewed. Patients of age less than 18 years were excluded from the study. Patients’ records were searched from January 2017 to December 2018. Data were retrieved for patients’ age (in years), gender, height (in cm), weight (in kg), primary complaint, diagnosis, and any comorbidity. BMI was calculated by dividing weight (in kg) by squared height (in m^2^). The Asian-specific cutoffs for BMI were used for categorizing patients as overweight (23-24.9 kg/m^2^) or obese (≥25 kg/m^2^) [15]. Comorbidity was observed on the basis of self-reported patient’s history, use of medicine for the underlying diseases, and/or confirmation of diagnosis during their follow-up visit in the clinic. Diabetes and impaired glucose tolerance were diagnosed using criteria defined by the American Diabetes Association. The criteria of the American Heart Association were used to diagnose dyslipidemia and HTN. Metabolic syndrome was identified based on the definition of the International Diabetes Federation. The American Thyroid Association guidelines were used for diagnosing thyroid disorders. Sub-fertility was diagnosed for patients who gave self-reported history, were either referred from sub-fertility clinic, or married for at least a year and had not conceived.

Data were entered into SPSS version 21 (IBM Corp., Armonk, NY, USA) for the purpose of analysis. Frequency and percentages were computed for categorical variables. Median with interquartile range (IQR) was calculated to present age and BMI after assessing the assumption of normality using the Shapiro-Wilk test. The chi-square linear-by-linear association test was applied to assess whether there was an increasing trend in disease frequency with increasing BMI. Logistic regression model was run to compute odd ratios and their 95% confidence interval to determine BMI as a predictor of NCDs. A two-tailed p-value of less than 0.05 was taken as statistically significant on logistic regression model.

## Results

A total of 613 patient records were retrieved and analyzed. The median age was 38 years (IQR=18-80 years) and BMI was 28.8 kg/m^2^ (IQR=24.6-33.05 kg/m^2^), respectively. Majority of the study participants were females (n=481 [70.6%]). Out of 613 patients, 103 (16.8%), 65 (10.6%), and 445 (72.6%) had a BMI of ≤22.9 kg/m^2^, 23-24.9 kg/m^2^, and ≥25 kg/m^2^, respectively. Among 510 (83.2%) people with elevated BMI (≥23 kg/m^2^), the most frequent NCDs were dyslipidemia (n=200 [39.2%]) followed by T2DM (n=166 [32.5%]), HTN (n=160 [31.4%]), thyroid disorders (n=146 [28.6%]) with 104 (71.2%) having hypothyroidism and the remaining 42 (28.8%) having hyperthyroidism, metabolic syndrome (n=127 [25%]), subfertility (n=76 [14.9%]), impaired glucose tolerance (n=65 [12.7%]), and autoimmune diseases (n=35 [6.9%]).

Data analysis showed an increasing trend in disease frequency with increasing BMI (Table 1), with the result being statistically significant for dyslipidemia (p<0.001), HTN (p<0.001), T2DM (p<0.001), impaired glucose tolerance (p<0.001), thyroid disorders (p=0.023), and metabolic syndrome (p<0.001). There was no statistically significant increase in autoimmune diseases (p=0.270) and subfertility (p=0.368) with increasing BMI. Age was found to be significantly associated with BMI, with a higher risk of obesity among people older than 39 years (p<0.001). There was no association of gender with BMI (p=0.053), as shown in Table 1.

**Table 1:** Distribution of different non-communicable diseases among patients with body mass index of ≤22.9, 23-24.9, and ≥25 kg/m2 †Pearson’s chi-square test is reported. *Significant at p<0.05. **Significant at p<0.01.

Study variables	Body mass index			p-Value
	≤22.9 kg/m^2^	23-24.9 kg/m^2^	≥25 kg/m^2^	
	n (%)	n (%)	n (%)	
Age (in years)				
18-29	54 (52.4)	15 (23.1)	104 (23.4)	<0.001†,**
30-39	26 (25.2)	18 (27.7)	110 (24.7)	
40-49	12 (11.7)	13 (20)	105 (23.6)	
50-59	4 (3.9)	13 (20)	82 (18.4)	
60 and above	7 (6.8)	6 (9.2)	44 (9.9)	
Gender				
Male	14 (13.6)	15 (23.1)	103 (23.1)	0.053†
Female	89 (86.4)	50 (76.9)	342 (76.9)	
Dyslipidemia				
Yes	10 (9.7)	20 (30.8)	180 (40.4)	<0.001**
No	93 (90.3)	45 (69.2)	265 (59.6)	
Hypertension				
Yes	6 (5.8)	17 (26.2)	143 (32.1)	<0.001**
No	97 (94.2)	48 (73.8)	302 (67.9)	
Diabetes				
Yes	12 (11.7)	17 (26.2)	149 (33.5)	<0.001**
No	91 (88.3)	48 (73.8)	296 (66.5)	
Impaired glucose tolerance				
Yes	0 (0)	7 (10.8)	58 (13)	<0.001**
No	103 (100)	58 (89.2)	387 (87)	
Thyroid disorders				
Yes	38 (36.9)	24 (36.9)	122 (27.4)	0.031*
No	65 (63.1)	41 (63.1)	323 (72.6)	
Autoimmune diseases				
Yes	11 (10.7)	4 (6.2)	31 (7)	0.270
No	92 (89.3)	61 (93.8)	414 (93)	
Metabolic syndrome				
Yes	0 (0)	7 (10.8)	120 (27)	<0.001**
No	103 (100)	58 (89.2)	325 (73)	
Subfertility				
Yes	21 (20.4)	9 (13.8)	67 (15.1)	0.368
No	82 (79.6)	56 (86.2)	378 (84.9)	

On logistic regression model when adjusted for age and gender, association between dyslipidemia and BMI was observed (Table 2). The risk of dyslipidemia increased by more than two-folds in overweight patients (OR=2.70; 95% CI: 1.12-6.52) and more than four-folds in obese patients (OR=4.38; 95% CI: 2.16-8.88) in contrast to the people having BMI ≤ 22.9 kg/m^2^. BMI was also found to be a predictor of HTN. The odds of HTN were higher in those who were overweight (OR=3.60; 95% CI: 1.27-10.22) or obese (OR=5.03; 95% CI: 2.08-12.16), in contrast to those who were of normal BMI. T2DM was also associated with BMI, with a higher risk of T2DM in patients who were obese (OR=2.15; 95% CI: 1.04-4.45).

**Table 2:** Risk of non-communicable diseases among overweight and obese Note: the model was adjusted for age and gender. †OR not computed as count in reference category was zero. *Significant at p<0.05. **Significant at p<0.01. IGT, impaired glucose tolerance

Non-communicable disease	BMI of 23-24.9 kg/m^2^, OR (95% CI)	p-Value	BMI of ≥25 kg/m^2^, OR (95% CI)	p-Value
Dyslipidemia	2.70 (1.12–6.52)	0.027	4.38 (2.16–8.88)	<0.001**
Hypertension	3.60 (1.27–10.22)	0.016*	5.03 (2.08–12.16)	<0.001**
Diabetes mellitus	1.32 (0.51–3.37)	0.566	2.15 (1.04–4.45)	0.038*
Thyroid disorders	1.02 (0.56–2.14)	0.798	0.69 (0.43–1.11)	0.127
Autoimmune diseases	0.50 (0.15–1.67)	0.259	0.56 (0.26–1.21)	0.139
Subfertility	1.10 (0.63–1.93)	0.728	0.73 (0.29–1.84)	0.502
IGT†	-	-	-	-
Metabolic syndrome†	-	-	-	-

Logistic regression model was run to determine the risk of NCDs among male and female cohorts of overweight/obese patients in contrast to the patients with normal BMI, and the model was adjusted for age (Table 3). There was no association of BMI with thyroid disorders, autoimmune diseases, and subfertility in both genders. A significant impact of BMI was observed in increasing the risk of dyslipidemia and HTN in males. In the male cohort, the risk of dyslipidemia and HTN was 7.40 times (OR=7.40; 95% CI: 1.50-36.58) and 9.33 times (OR=9.33; 95% CI: 1.13-77.23) higher in obese patients, respectively, in contrast to the patients with normal BMI. Among females, BMI was identified as an independent risk factor for dyslipidemia, HTN, and T2DM. In the female cohort, the odds of dyslipidemia were higher in obese females (OR=3.99; 95% CI: 1.82-8.79) than in females with normal BMI. The risk of HTN was significantly higher in overweight (OR=4.52; 95% CI: 1.38-14.87) and obese women (OR=4.75; 95% CI: 1.75-12.91). The odds of T2DM were 2.50 times higher (OR=2.5; 95% CI: 1.04-6.0) in obese females as compared to females with normal BMI.

**Table 3:** Risk of non-communicable diseases among overweight and obese for male and female cohort Note: the model was adjusted for age. †OR not computed as count in reference category was zero. *Significant at p<0.05. **Significant at p<0.01. IGT, impaired glucose tolerance

Non-communicable disease	BMI of 23-24.9 kg/m^2^, OR (95% CI)	p-Value	BMI of ≥25 kg/m^2^, OR (95% CI)	p-Value
Male cohort				
Dyslipidemia	5.79 (0.89–37.53)	0.066	7.40 (1.50–36.58)	0.014*
Hypertension	3.24 (0.30–35.56)	0.335	9.33 (1.13–77.23)	0.038*
Diabetic mellitus	0.92 (0.17–5.00)	0.920	1.42 (0.36–5.60)	0.614
Thyroid disorders	1.80 (0.13–24.15)	0.657	2.69 (0.31–23.19)	0.369
Autoimmune diseases	1.32 (0.07–26.11)	0.855	1.62 (0.18–14.55)	0.665
Subfertility	1 (0.06–16.26)	0.99	0.57 (0.09–3.53)	0.548
IGT	-	-	-	-
Female cohort				
Dyslipidemia	2.14 (0.77–5.95)	0.146	3.99 (1.82–8.79)	0.001**
Hypertension	4.52 (1.38–14.87)	*0.013	4.75 (1.75–12.91)	0.002**
Diabetic mellitus	1.50 (0.48–4.71)	0.489	2.50 (1.04–6.0)	0.040*
Thyroid disorders	1.13 (0.56–2.29)	0.738	0.63 (0.38–1.03)	0.065
Autoimmune diseases	0.44 (0.11–1.72)	0.238	0.46 (0.20–1.05)	0.064
Subfertility	0.68 (0.25–1.82)	0.446	1.18 (0.65–2.13)	0.584
IGT†	-	-	-	-
Metabolic syndrome†	-	-	-	-

## Discussion

This study determined the prevalence of elevated BMI (≥23 kg/m^2^) and the association of BMI with the NCDs in this group. Multiple investigators from Western countries and other Asian countries have reported on the prevalence of obesity and its association with comorbidities; however, our local data are scarce [16,17]. A study from Peshawar had determined the association of NCDs with obesity, but the study only included female subjects [18].

In this study, we found a high prevalence for elevated BMI (83.2%). A previously published study from Karachi reported 21% study participants being obese [19]. Another study conducted in Balochistan reported 37% participants as being either overweight (15.3%) or obese (19.5%) [20]. A household survey reported from urban households of Karachi revealed 35.2% and 33.1% prevalence of overweight and obesity, respectively [21]. The high prevalence of obesity in our study cohort is in all probability due to the fact that these patients were attending an endocrine clinic for obesity and its related diseases.

The prevalence of overweight/obesity was not significantly different between the two genders in this study, as has also been reported by the WHO estimates of 2016 [2]. However, a Chinese study reported the prevalence of overweight to be higher in men than women, while obesity prevalence was not significantly different (27.2% versus 25.8%) [22]. The same finding was depicted in a Russian study that showed a considerably large difference in the prevalence of overweight between men and women (42.3% in men and 28.7% in females), whereas there was a small difference in obesity prevalence between males and females (27.5% in men versus 31.4% in women) [23]. The equal occurrence of overweight and obesity in our cohort could be due to the disproportionately smaller number of male patients.

In our study, the most prevalent NCD among people with elevated BMI was dyslipidemia (39.2%) followed by T2DM (32.5%), HTN (31.4%), thyroid disorders, metabolic syndrome, subfertility, impaired glucose tolerance, and autoimmune diseases. In contrast, a Chinese study reported the highest frequency of association with HTN (46.6%) followed by dyslipidemia (35%) and T2DM (7.1%) among obese participants [22]. In a study from West Texas, among patients with elevated BMI, the highest prevalence was observed for HTN (67%) followed by dyslipidemia (59.1%), T2DM (46.8%), OSA (19.5%), coronary artery disease (20.1%), and myocardial infarction (6.3%) [17]. Despite the difference in underlying disease prevalence and ranking, the findings of our study were similar to other studies in terms of the top three prevalent diseases, which are dyslipidemia, HTN, and T2DM. In our study, a very small number of patients spontaneously complained of symptoms suggestive of OSA, whereas the prevalence of OSA has been reported as 40% to 90% in patients with severe obesity (BMI > 40 kg/m^2^) [24]. In addition to HTN, T2DM, and dyslipidemia, our study also reported a frequency of thyroid disorders, metabolic syndrome, autoimmune diseases, subfertility, and impaired glucose tolerance, but these were not significantly associated with BMI. Contrary to our finding, these diseases have been reported to be associated with BMI in other studies [25-30]. This highlights the fact that a large community-based research should be conducted to simultaneously correlate obesity with all NCDs.

Although our study included a larger sample than previously conducted local investigations and also studied obesity and NCDs concurrently, it presented the data of patients visiting an endocrinology clinic and hence the estimated prevalence of obesity and overweight is not representative of the prevalence in the general population. Since the study was retrospective in nature, the educational and socioeconomic status of study participants could not be retrieved, which are key confounders. Hence, we suggest to replicate this study in the general population incorporating all the stated limitations to accurately determine the current burden of overweight/obesity in our society and its association with NCDs.

## Conclusions

This study demonstrated a higher prevalence of obesity in patients visiting a private endocrinology clinic. Obesity was identified as an independent risk factor for dyslipidemia, HTN, and diabetes. Future studies are suggested to determine the burden of obesity in the general population and establish its association with all likely NCDs.
